# Prediction of Ventricular Arrhythmias by QRS/QTc - Ratio in Citalopram or Escitalopram Intoxication

**DOI:** 10.3389/fmed.2022.866454

**Published:** 2022-03-16

**Authors:** Erik Sveberg Dietrichs, Godfrey L. Smith

**Affiliations:** ^1^Experimental and Clinical Pharmacology Research Group, Department of Medical Biology, UiT, The Arctic University of Norway, Tromsø, Norway; ^2^Center for Psychopharmacology, Diakonhjemmet Hospital, Oslo, Norway; ^3^Institute of Cardiovascular and Medical Sciences, University of Glasgow, Glasgow, United Kingdom

**Keywords:** citalopram, escitalopram, long QT, ECG, QRS/QTc, arrhythmia, ventricular fibrilation, torsade de pointes (TdP)

## Abstract

**Background:**

The U.S. Food and Drug Administration (FDA) has stated that citalopram and escitalopram should not be used at daily doses above 40 mg/20 mg due to risk for development of fatal ventricular arrhythmias like torsade de pointes (TdP). Yet, supratherapeutic serum concentrations of citalopram are common and predicting patients at risk for TdP is of high clinical value. Accordingly, we investigated whether QRS/QTc; developed for predicting TdP in hypothermic patients could be used in citalopram intoxication.

**Methods:**

A total of 16 publications describing patients suffering from complications due to citalopram or escitalopram treatment, or intoxication with the same substances, were included after a systematic search. The main criterion for inclusion was admission ECG, either with given QRS and QTc values or with attached ECG-files that enabled calculation.

**Results:**

QRS/QTc rather that QTc alone emerged as a marker of ventricular arrhythmia in the 16 included case reports, with highly significant (*p* < 0.0005) lower values in patients displaying ventricular arrhythmias.

**Conclusion:**

Citalopram and escitalopram are extensively used in treatment of depressive disorders, and a large proportion of patients have supratherapeutic serum concentrations. Calculation of QRS/QTc in available case reports show that this novel ECG-marker has potential to predict patients at risk for developing ventricular arrhythmias.

## Background

In 2011, The U.S. Food and Drug Administration (FDA) issued a Drug Safety Communication stating that the selective serotonin reuptake inhibitor (SSRI) citalopram should no longer be used at daily doses above 40 mg, due to risk of QT prolongation and fatal development of ventricular arrhythmias, like torsade de pointes (TdP) (1). Underlining the FDA recommendations, both citalopram and its (S)-stereoisomer escitalopram was found to increase mortality risk compared to other anti-depressants, related to elevated incidence of ventricular arrhythmias in citalopram-users (2). Accordingly also escitalopram-doses are recommended to be restricted to a maximal daily dose of 20 mg (1).

Yet, escitalopram is extensively used in patients suffering from conditions such as depression and anxiety-disorders. More than 2% of the Norwegian population were prescribed escitalopram in 2020, making it both the most used SSRI and antidepressant drug in the Norwegian population (3). Generally, escitalopram in therapeutic doses is well tolerated but the high volume of users advocates the need for a tool to determine risk for adverse cardiac events. Predicting which patients that will develop such arrhythmias remains a significant clinical challenge. QTc alone does not give sufficient information to predict pro-arrhythmic activity in escitalopram-users and accordingly there is no well-established ECG value for assessing the risk of TdP (4).

QTc-prolongation with increased risk for TdP and cardiac arrest is a known complication in treatment with several psychopharmacological substances, but is relevant also in other patients groups and conditions like hypothermia (5). From pre-clinical and clinical data, we have recently developed the ECG-marker QRS/QTc that could predict risk for ventricular fibrillation (VF) and cardiac arrest with low core temperatures (6, 7). It is important to assess whether our findings also apply to predicting risk for developing ventricular arrhythmias in other patient groups with QTc-prolongation. Accordingly, intoxication with citalopram or escitalopram is a relevant condition. Successful identification of intoxicated patients at risk for developing ventricular arrhythmias could provide opportunity to initiate preventive pharmacological treatment before life-threatening arrhythmias arise. Accordingly, we have assessed case-reports describing emergency admission due to treatment or intoxication with either citalopram or escitalopram, where patients were undergoing electrophysiological monitoring with ECG-recordings.

## Methods

A literature search was conducted on April 13th, 2020 in the electronic PubMed database. All case reports retrieved for: #1 citalopram AND QRS AND QTC, #1 escitalopram AND QRS AND QTC, #3 citalopram AND cardiac arrest, #4 escitalopram AND cardiac arrest, #5 citalopram AND torsades de pointes, #6 escitalopram AND torsades de pointes, #7 citalopram AND overdose, and #8 escitalopram AND overdose, were assessed.

A total of 16 publications were included (8–23). The main criterion for inclusion of articles was that they had an admission ECG, either with given QRS and QTc values or with attached ECG-files that enabled us to calculate these parameters. Only case reports describing acute admission of patients suffering from complications due to citalopram or escitalopram treatment, or intoxication with the same substances, were included. Case reports lacking an admission ECG and case reports on children and neonates, were excluded. One case report with an admission ECG was excluded due to that this ECG was taken during monomorphic ventricular tachycardia, leaving us unable to assess ECG-timings prior to ventricular arrhythmia (24).

Studies were considered for inclusion based on the contents of the abstract. If this was inadequate or absent, the full text was assessed to examine whether they met inclusion criteria. Articles that were not detected through the literature search were found in reference-lists of included manuscripts or other literature reviews.

All data were normally distributed (assessed with Shapiro-Wilk’s test) and passed a Brown-Forsythe test of equal variance. To assess differences between patients that did or did not experience ventricular arrhythmias, a two-tailed, unpaired *t*-test was used. Results are presented as mean ± Standard deviation. Differences were considered significant at *p* < 0.05.

## Results

A total of 16 case reports, concerning 11 female and five male patients aged 20–89 (mean 43) were included (Table 1). Twelve were admitted to hospital after complications related to intoxication with citalopram (*n* = 9) (dose range: 0.22–11.6 g) or escitalopram (*n* = 3) (0.14–0.4 g). Four patients were admitted due to serious side effects of using therapeutic doses of citalopram (0.08–0.04 g). Twelve of the patients had taken additional drugs or ethanol, either as part of their prescribed treatment regime or due to voluntary intoxication. Serum concentrations were available for five of the citalopram intoxications (mean 2,827 ng/mL) and one escitalopram intoxication (290 ng/mL).

**TABLE 1:** Clinical data from the included patients, as given in the case-reports.

Case report	Age	Sex	Type of poisoning	Substance	Serum concentration (ng/mL)	Dose (g)	Other drugs	VT/VF
(1) Bin Salih et al. (19)	20	F	Intoxication	Citalopram	–	1.8	Ethanol	No
(2) Lung et al. (15)	21	M	Intoxication	Citalopram	522	11.6	Olanzapine Clonazepam	No
(3) Venkatraman et al. (20)	23	F	Intoxication	Citalopram	–	0.22	Lamotrigine Chlorphenamine Ethanol	No
(4) Cuenca et al. (22)	23	M	Intoxication	Citalopram	–	0.92	No	No
(5) Lung et al. (9)	24	F	Intoxication	Citalopram	400	1.8	Bupropion Clonazepam	Yes
(6) Farkas et al. (17)	25	F	Intoxication	Escitalopram	290	-	Lamotrigine	No
(7) Engebretsen et al. (16)	31	M	Intoxication	Citalopram	1,940	0.4	Ethanol	No
(8) Mohammed et al. (18)	33	F	Intoxication	Escitalopram	–	0.4	Lithium	No
(9) Kraai et al. (23)	35	F	Intoxication	Citalopram	7,300	–	Cannabis	Yes
(10) Deshmukh et al. (11)	40	F	Therapeutic dose	Citalopram	–	0.08	No	Yes
(11) Gregorio et al. (12)	48	F	Therapeutic dose	Citalopram	–	0.04	Furosemide	Yes
(12) Baranchuk et al. (21)	52	M	Intoxication	Escitalopram	–	0.14	Diazepam Zopiclone	No
							Lorazepam	
							Morphine	
(13) Liotier et al. (14)	54	F	Intoxication	Citalopram	–	–	Ethanol	Yes
(14) Kanjanauthai et al. (10)	81	M	Therapeutic dose	Citalopram	–	–	–	Yes
(15) Brucculeri et al. (8)	82	F	Intoxication	Citalopram	910	1.6	No	No
(16) Agosti et al. (13)	89	F	Therapeutic dose	Citalopram	–	0.04	Levosulpiride	Yes
Average ± SD	43 ± 23	–	–	–	2,464 ± 2,687	1.59 ± 3.09	–	–

A total of seven included patients had ventricular arrhythmias after admission and two of these died after final ECG-readings of pulseless tachycardia. In 5 of 7 patients, the ventricular arrhythmia was described as TdP. In the remaining nine patients, not developing ventricular arrhythmias, one went into sinus arrest after readings of increased QRS-widening but recovered and had no sequela. Other patients in this group also experienced sinus tachycardia, bradycardia or left bundle branch block during hospitalization, and the majority (6 of 9) were unconscious or experienced seizures at or after admission.

**FIGURE 1:**
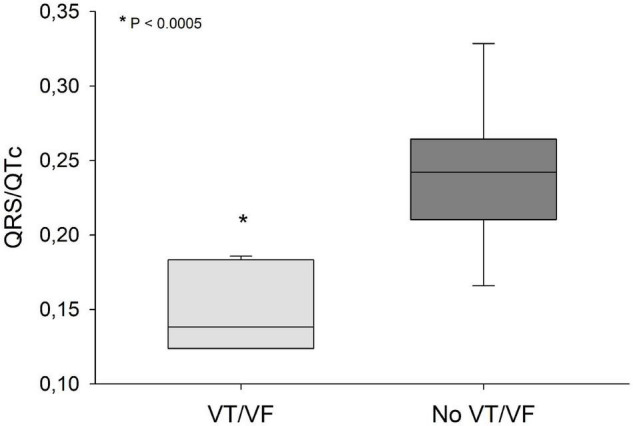
QRS/QTc values were significantly lower (*p* < 0.0005) in patients that developed VT/VF after first ECG-recording compared to patients that remained in sinus rhythm. Solid line: Median, box-plot: 25- to 75-persentile, and error bars: 5- and 95-persentile. *Difference from «No VT/VF group» (*p* < 0.05). VF, ventricular fibrillation; VT, ventricular tachycardia.

**FIGURE 2:**
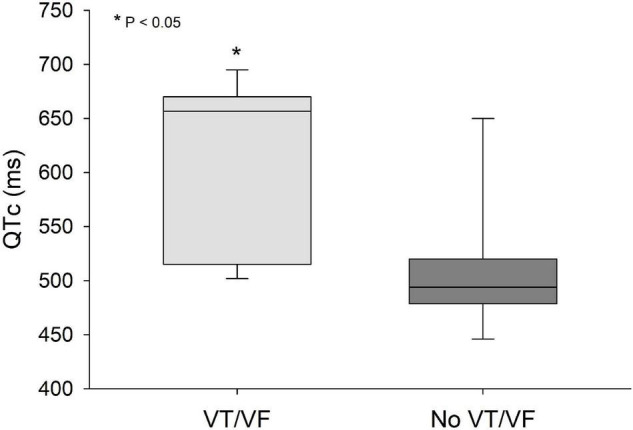
QTc values were significantly lower (*p* < 0.05) in patients that developed VT/VF after first ECG-recording compared to patients that remained in sinus rhythm. Solid line: Median, box-plot: 25- to 75-persentile, and error bars: 5- and 95-persentile. *Difference from «No VT/VF group» (*p* < 0.05). VF, ventricular fibrillation; VT, ventricular tachycardia.

**FIGURE 3:**
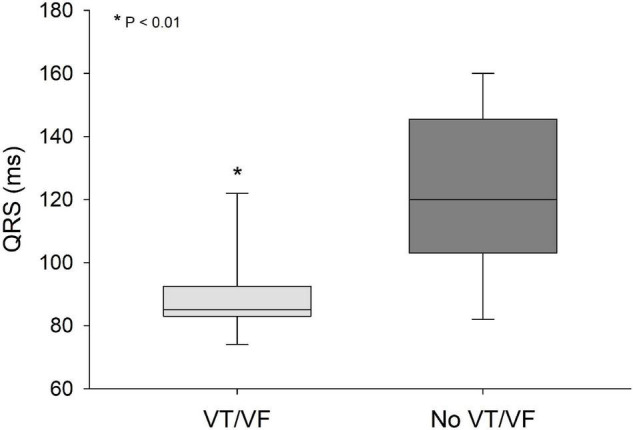
QRS values were significantly lower (*p* < 0.01) in patients that developed VT/VF after first ECG-recording compared to patients that remained in sinus rhythm. Solid line: Median, box-plot: 25- to 75-persentile, and error bars: 5- and 95-persentile. *Difference from «No VT/VF group» (*p* < 0.05). VF, ventricular fibrillation; VT, ventricular tachycardia.

**FIGURE 4:**
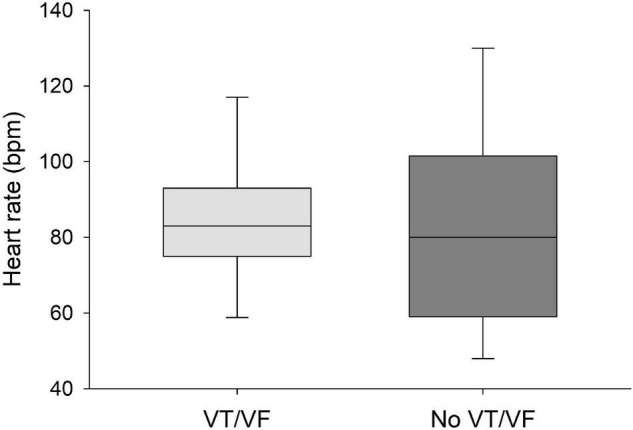
No significant differences were found in heart rate between patients that developed VT/VF after first ECG-recording and patients that remained in sinus rhythm. Solid line: Median, box-plot: 25- to 75-persentile, and error bars: 5- and 95-persentile.

QRS/QTc value emerged as the best indicator of ventricular arrhythmia (Figures 1–4 and Table 2) in the 16 included case reports, with highly significant lower values in the ventricular arrhythmia patient group (0.15 ± 0.02 vs. 0.24 ± 0.04, *p* < 0.0005). This difference was the most substantial between groups although both lower QRS values (90 ± 14 vs. 122 ± 23, *p* < 0.01) and longer QTc was found in the same patients (606 ± 78 vs. 508 ± 55, *p* < 0.05). There were no differences in heart rate (85 ± 17 vs. 81 ± 25).

**TABLE 2:** ECG-data from the included patients as given in the case-reports or calculated from included ECG-recordings.

Case report	Age	QRS (ms)	QT (ms)	QTc (ms)	HR (bpm)	QRS/QTc	VT/VF
(1) Bin Salih et al. (19)	20	82	428	494	80	0.17	No
(2) Lung et al. (15)	21	160	400	487	85	0.33	No
(3) Venkatraman et al. (20)	23	120	352	470	107	0.26	No
(4) Cuenca et al. (22)	23	108	446	446	60	0.24	No
(5) Lung et al. (9)	24	85	411	515	93	0.17	Yes
(6) Farkas et al. (17)	25	98	392	496	96	0.2	No
(7) Engebretsen et al. (16)	31	124	344	506	130	0.25	No
(8) Mohammed et al. (18)	33	117	479	491	63	0.24	No
(9) Kraai et al. (23)	35	92	360	502	117	0.18	Yes
(10) Deshmukh et al. (11)	40	74	357	535	90	0.14	Yes
(11) Gregorio et al. (12)	48	83	620	670	75	0.12	Yes
(12) Baranchuk et al. (21)	52	145	727	650	48	0.22	No
(13) Liotier et al. (14)	54	83	600	670	75	0.12	Yes
(14) Kanjanauthai et al. (10)	81	92.5	572	695	83	0.13	Yes
(15) Brucculeri et al. (8)	82	146	544	534	58	0.27	No
(16) Agosti et al. (13)	89	122	650	657	59	0.19	Yes
Average ± SD	43 ± 23	108 ± 25	480 ± 118	551 ± 82	82 ± 22	0.20 ± 0.06	–

## Discussion

Prolongation of the QT-interval is related to treatment with several psychopharmacological substances. The underlying challenge is to detect patients that are at risk for developing ventricular arrhythmias, like TdP, and take actions to reduce this risk. Here we present data advocating that the novel ECG-biomarker QRS/QTc has potential to predict predisposition to such arrhythmias and therefore serve as an ECG-marker of increased risk for cardiac arrest.

In recent publications we have found indication that QRS/QTc values below 0.2 were associated with increased risk for hypothermic cardiac arrest (5–7). The effects of hypothermia on the electrophysiology of the human heart are similar to those of citalopram- or escitalopram and other agents used to treat psychiatric disorders that prolong repolarization. While severe accidental hypothermia is uncommon, use of antidepressants is widespread. In Norway more than 6% of the population was prescribed antidepressant drugs in 2020 (3), with even higher numbers in other European countries. A recent Spanish study showed that 8.6% of the general population used antidepressants (25). Greater age is associated with higher risk of cardiac disease, which could be complicated by more extensive use of drugs. Eleven percent of Norwegian senior residents are prescribed antidepressant drugs, with citalopram and escitalopram being among the most common substances. Twelve percent of users had serum concentrations of citalopram above the recommended reference range, with increasing risk of adverse effects (26).

In isolated guinea-pig cardiomyocytes it has been shown that citalopram inhibits the expression of hERG-channels available to generate repolarizing K^+^-current, with an IC_50_ of 12.2 ng/mL (27). This concentration is more than 200-fold lower than the average serum concentration from 5 of the citalopram-intoxicated patients included in the present study, although direct comparison is difficult to due significant plasma protein binding of the drug. At higher concentrations, citalopram inhibits L-type Ca^2+^ channels (LTCC) (27). It has been advocated that citalopram-induced blockade of LTCCs mitigate the potential adverse effects of hERG-inhibition, by preventing early after depolarizations (EAD) and subsequent arrhythmias like TdP (28). The linking of anti-arrhythmic effects to QRS duration may be based in the ability of citralopram to block the Nav1.2 voltage gated sodium channel (VGSC) (29). The accompanying reduced myocardial excitability and potentially conduction velocity may contribute to the anti-arrhythmic properties under the same principle as Vaughan-Williams Class I anti-arrhythmic drugs (30). A similar logic can be used to explain the anti-arrhythmic effects of profound hypothermia where both QRS and QT are increased (5–7, 31). A mild reduction of excitability by inhibition of the VGSC and the associated reduced TdP-risk would not be detected by monitoring QTc-interval, which is prolonged predominately by effects on repolarization. Although long QT-interval is a common complication of citalopram- and other psychopharmacological treatments, to-date no reliable marker of arrhythmia-risk exists in these patients.

Given that citalopram-induced blockade of VGSC and LTCC may mitigate EAD-risk caused by hERG-inhibition, the risk of drug-induced arrhythmias due to hERG-blockade remains the dominant effects, as the IC_50_-values of citalopram for inhibiting hERG is 10-fold lower than for inhibition of VGSC and LTCC (28). Individuals exposed to high concentrations of citalopram in the range above the threshold for hERG-inhibition and below VGSC channel-inhibition, could theoretically be at higher risk for developing TdP. QRS/QTc values would be low in such patients as opposed to higher QRS/QTc values in patients with the co-occurrence of reduced excitability/conduction velocity (prolonged QRS) and delayed repolarization (prolonged QTc).

Citalopram and escitalopram are mainly subject to hepatic metabolism by CYP2C19. CYP2C19-mutations that reduce metabolism could therefore explain high serum concentrations (26) of the mother compound (escitalopram/citalopram) compared to the metabolite demethylcitalopram in some patients. Other metabolites include didemethylcitalopram via CYP2D6 metabolism. Neither of these metabolites contribute to the pharmacologic activity, and normally exist in the plasma in small quantities. Although not contributing to the antidepressant effect of citalopram or escitalopram, demethylcitalopram and didemethylcitalopram are electrophysiologically active metabolites, inhibiting both the IKr and IKs potassium channels (32), theoretically reducing QRS/QTc values. Didemethylcitalopram appears the most cardiotoxic and has been associated with unexpected and sudden death in dogs (33), probably due to species-specific metabolic differences as humans generally have lower didemethylcitalopram-concentrations (32). Nevertheless, some patients appear to have high didemethylcitalopram serum-concentrations after therapeutic escitalopram-doses at 20 mg or less per day (34). Drug interactions as well as genetic variation of CYP2C19 and CYP2D6 could therefore alter serum concentrations of citalopram metabolites and cause individual differences in cardiac toxicity of citalopram and escitalopram. Therapeutic drug monitoring would not reveal patients at such risk, unless the metabolites are monitored as well as citalopram concentrations. Accordingly, QRS/QTc values could potentially reveal patients with CYP2C19 and CYP2D6 metabolism that biases these patients toward pro-arrhythmic activity when using escitalopram or citalopram.

Use of antidepressants is also common in intended intoxications. Recently, suicide rates among older women in the United States has been increasing, and toxicology reports show that 47% of overdoses were positive for antidepressants (35). Accordingly, both unintentional and intentional citalopram-intoxications are relevant conditions both for general practitioners, psychiatrists and in emergency medicine. An ECG-marker more reliable than QTc for detecting risk of TdP in exposed patients would be of high clinical value, both in the emergency room after intoxications and as a simple screening tool in patients with citalopram or escitalopram prescriptions.

Prolongation of QT-interval and increased risk of TdP, triggered by drugs, is termed “acquired long QT syndrome” (aLQTS) (36). In vulnerable patients, with underlying genetic substrates, aLQTS is triggered more easily. A third of patients that present with aLQTS have congenital LQTS mutations (36). Accordingly, close monitoring is necessary to prevent unexpected death due to QT-prolongation and TdP, during treatment with psychopharmaca. Currently there is no well-established ECG value for assessing the risk of TdP (4). To speculate; the relation we find between QRS/QTc-values and VF in citalopram and escitalopram-intoxication could be due to undetected, congenital LQTS mutations. Accordingly, there is potential that QRS/QTc could serve as an inexpensive tool to detect underlying genetic disposition for aLQTS, both in intoxications and therapeutic use of drugs known to increase risk for TdP.

## Limitations

The current systematic review of case-reports cannot reveal possible underlying mechanisms that could explain which patients that develop TdP and cardiac arrest after intoxication with escitalopram or citalopram. Such mechanisms could include LQTS mutations but also interaction with or simultaneous intoxication with other drugs that were not detected in the included cases and that could have affected QRS/QTc-values.

## Conclusion

Citalopram and escitalopram are extensively used in treatment of depressive disorders, and a large proportion of patients have serum concentrations that exceed recommended reference intervals for safe treatment. Ventricular arrhythmias like torsades des pointes and cardiac arrest is a potential and life-threatening complication of such supratherapeutic serum concentrations. Calculation of QRS/QTc in available case reports show that this novel ECG-marker has potential to predict patients at risk for developing ventricular arrhythmias.

## Data Availability Statement

The original contributions presented in the study are included in the article/supplementary material, further inquiries can be directed to the corresponding author.

## Author Contributions

ED carried out the systematic search and screened case-reports for inclusion. ED and GS evaluated the data, wrote the manuscript, contributed to the article, and approved the submitted version.

## Conflict of Interest

The authors declare that the research was conducted in the absence of any commercial or financial relationships that could be construed as a potential conflict of interest.

## Publisher’s Note

All claims expressed in this article are solely those of the authors and do not necessarily represent those of their affiliated organizations, or those of the publisher, the editors and the reviewers. Any product that may be evaluated in this article, or claim that may be made by its manufacturer, is not guaranteed or endorsed by the publisher.
